# *QuickStats:* Percentage Distribution* of Adult Day Services Centers,^†^ by Type of Service^§^ — National Study of Long-Term Care Providers, 2016^¶^

**DOI:** 10.15585/mmwr.mm6732a8

**Published:** 2018-08-17

**Figure:**
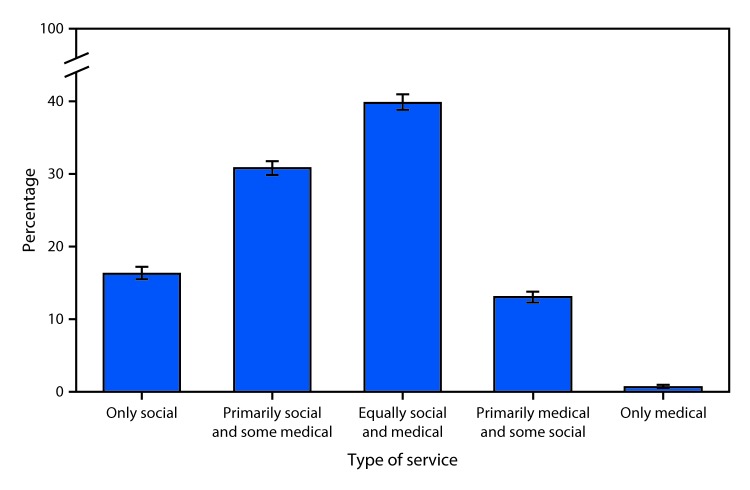
In 2016, four in 10 adult day services centers had services that were designed to meet both the social and medical needs of their enrolled participants equally. Approximately 31% of adult day services centers had services to meet primarily social needs and some medical needs of participants, 16% had services to meet only social needs, 13% had services to meet primarily medical needs and some social needs, and 1% had services to meet only medical needs.

**Source: **National Study of Long-Term Care Providers, 2016. https://www.cdc.gov/nchs/nsltcp/index.htm.

